# μ-2,2′-Bipyrimidine-κ^4^ *N* ^1^,*N* ^1′^:*N* ^3^,*N* ^3′^-bis­[iodido(triphenyl­phosphane-κ*P*)copper(I)] dimethyl­formamide disolvate

**DOI:** 10.1107/S1600536812028139

**Published:** 2012-06-30

**Authors:** Mohammed Fettouhi

**Affiliations:** a Department of Chemistry, King Fahd University of Petroleum and Minerals, Dhahran 31261, Saudi Arabia

## Abstract

In the title binuclear centrosymmetric complex, [Cu_2_I_2_(C_8_H_6_N_4_)(C_18_H_15_P)_2_]·2C_3_H_7_NO, the bis-bidentate 2,2′-bipyrimidine ligand bridges two copper(I) ions, each additionally bound to an iodide anion and a triphenyl­phosphane ligand in a distorted tetra­hedral N_2_IP geometry. The complex mol­ecules pack in columns parallel to [100] generating cavities occupied by dimethyl­formamide solvent mol­ecules. Weak C—H⋯I hydrogen-bonding inter­actions help to stabilize the crystal packing.

## Related literature

For copper(I) mixed-ligand complexes based on diimines and phosphanes, see: Costa *et al.* (2011 ▶); Fazal *et al.* (2009 ▶). For 2,2′-bipyrimidine polymetallic complexes, see: Albores & Rentschler (2009 ▶); Yucesan *et al.* (2009 ▶). For the analogous chlorido complex, see: Tan *et al.* (2012 ▶).
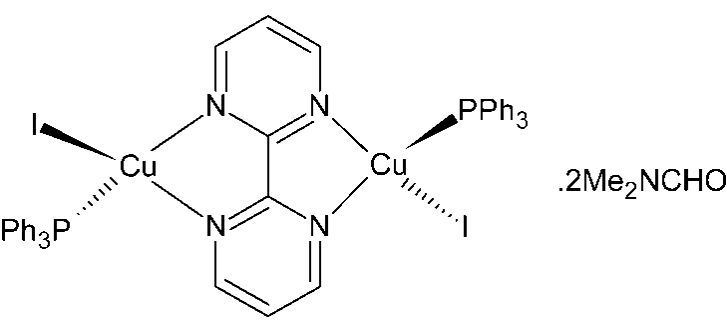


## Experimental

### 

#### Crystal data

[Cu_2_I_2_(C_8_H_6_N_4_)(C_18_H_15_P)_2_]·2C_3_H_7_NO
*M* *_r_* = 1209.78
Monoclinic, 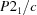

*a* = 9.2436 (5) Å
*b* = 14.0911 (8) Å
*c* = 20.1932 (11) Å
β = 92.232 (1)°
*V* = 2628.2 (3) Å^3^
*Z* = 2
Mo *K*α radiation
μ = 2.09 mm^−1^
*T* = 298 K
0.70 × 0.17 × 0.14 mm

#### Data collection

Bruker SMART APEX CCD diffractometer
Absorption correction: multi-scan (*SADABS*; Sheldrick, 1996 ▶) *T*_min_ = 0.323, *T*_max_ = 0.759
35525 measured reflections
6544 independent reflections
4457 reflections with *I* > 2σ(*I*)
*R*_int_ = 0.031

#### Refinement

*R*[*F* ^2^ > 2σ(*F* ^2^)] = 0.044
*wR*(*F* ^2^) = 0.114
*S* = 1.02
6544 reflections
291 parameters
H-atom parameters constrained
Δρ_max_ = 1.15 e Å^−3^
Δρ_min_ = −0.72 e Å^−3^

### 

Data collection: *SMART* (Bruker, 2007 ▶); cell refinement: *SAINT* (Bruker, 2007 ▶); data reduction: *SAINT*; program(s) used to solve structure: *SHELXS97* (Sheldrick, 2008 ▶); program(s) used to refine structure: *SHELXL97* (Sheldrick, 2008 ▶); molecular graphics: *ORTEP-3* (Farrugia, 1997 ▶); software used to prepare material for publication: *publCIF* (Westrip, 2010 ▶).

## Supplementary Material

Crystal structure: contains datablock(s) I, global. DOI: 10.1107/S1600536812028139/wm2646sup1.cif

Structure factors: contains datablock(s) I. DOI: 10.1107/S1600536812028139/wm2646Isup2.hkl

Additional supplementary materials:  crystallographic information; 3D view; checkCIF report

## Figures and Tables

**Table:** 

Cu1—N3	2.075 (3)
Cu1—N4^i^	2.155 (3)
Cu1—P1	2.1857 (10)
Cu1—I1	2.5731 (6)

**Table:** 

N3—Cu1—N4^i^	78.97 (10)
N3—Cu1—P1	124.71 (9)
N4^i^—Cu1—P1	119.53 (8)
N3—Cu1—I1	105.04 (9)
N4^i^—Cu1—I1	100.92 (8)
P1—Cu1—I1	119.15 (3)

Symmetry code: (i) 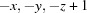.

**Table 2:** Hydrogen-bond geometry (Å, °)

*D*—H⋯*A*	*D*—H	H⋯*A*	*D*⋯*A*	*D*—H⋯*A*
C4—H4⋯I1^ii^	0.93	3.03	3.796 (4)	140

Symmetry code: (ii) 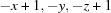.

**Fig. 1.**
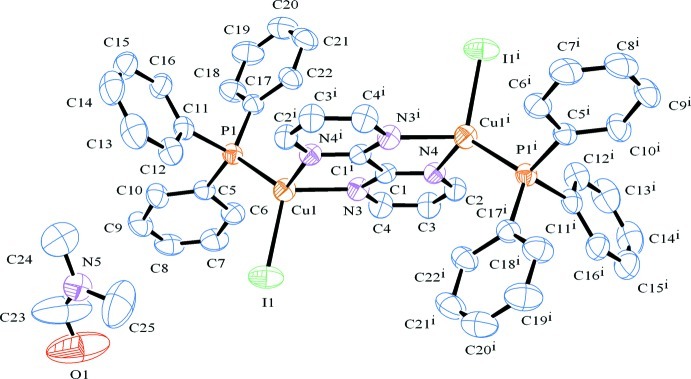
Molecular structure of the title compound showing the atomic numbering scheme. Displacement ellipsoids are drawn at the 30% probability level (Symmetry code: i = -x,-y,-z + 1).

**Fig. 2.**
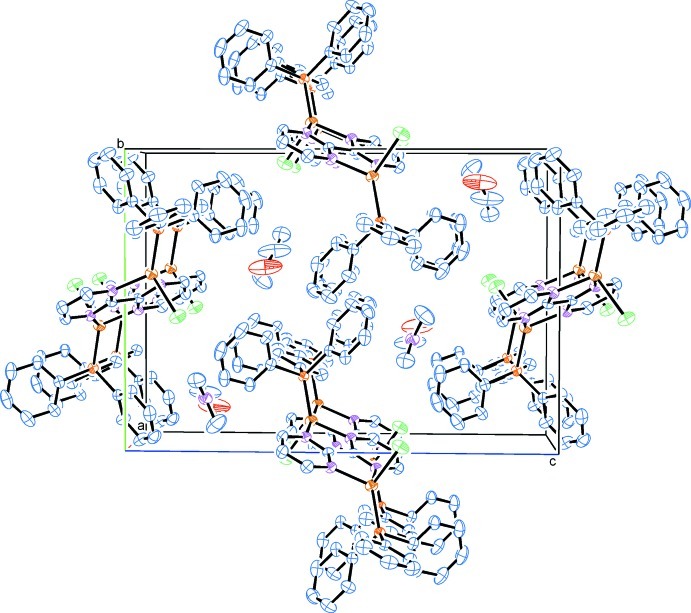
The packing of the structure of (I).

## supplementary crystallographic information

### Comment

Copper(I) mixed-ligand complexes based on diimines and phosphanes exhibit attracting photophysical and catalytic properties (Costa *et al.*, 2011; Fazal *et al.*, 2009). The 2,2'-bipyrimidine ligand (bpm) is of interest owing to its bis-chelating coordination ability, allowing the design of one-, two- and three-dimensional polymeric solids with interesting chemical and physical properties (Albores & Rentschler, 2009; Yucesan *et al.*, 2009). Herein is reported on a bimetallic mixed-ligand copper(I) iodido complex based on 2,2'-bipyrimidine and triphenylphosphane, [Cu_2_I_2_{P(C_6_H_5_)_3_}_2_(C_8_H_6_N_4_)]^.^2(C_3_H_7_NO), (I).

The asymmetric unit of (I) contains one half-molecule of the complex [Cu_2_I_2_(Ph_3_P)_2_(bpm)] and one dimethylformamide solvent molecule. The complete complex is generated by inversion symmetry with the inversion centre being located on the central C—C bond of the bipyrimidine ligand. One bis-chelating bpm ligand bridges two Cu(I) ions which are each additionally bound to an iodide anion and a phosphorus atom of the phosphane ligand. The geometry around the metal ion is distorted tetrahedral (Figure 1). The triphenylphosphane *ipso* carbon atoms and the iodide anion adopt an *anti* configuration with respect to the rotation around the Cu—P bond with a torsion angle (C17—P1—Cu1—I1) of -163.2 (1) °. The unfavorable *syn* configuration is observed in the analogous chlorido complex reported recently which is likely stabilized by intra-molecular π—π interactions (Tan *et al.*, 2012). The molecules of the complex (I) pack in columns parallel to [100] generating cavities occupied by the solvent molecules (Figure 2). Weak C—H···I hydrogen bonding interactions help to stabilize the crystal packing.

### Experimental

CuI (1.0 mmol, 0.1904 g), Ph_3_P (1.0 mmol, 0.2623 g) and 2,2'-bipyrimidine (0.5 mmol, 0.0790 g) were reacted in dimethylformamide (35 ml) at 338 K for 2 h. The red solution was then filtered. After a few days, red crystals suitable for X-ray diffraction were obtained by slow evaporation.

### Refinement

All H atoms were placed in calculated positions with C—H distances of 0.93 Å (*sp*^2^ carbon atoms), or 0.96 Å (*sp*^3^ carbon atoms). The isotropic displacement parameters were *U*_iso_(H) = 1.5*U*_eq_(C) for the methyl atoms and *U*_iso_(H) = 1.2*U*_eq_(C) for all other atoms. The highest remaining electron density is located 0.97 Å from atom I1, and the lowest electron density 0.88 Å from the same atom.

### Crystal data

**Table:**

[Cu_2_I_2_(C_8_H_6_N_4_)(C_18_H_15_P)_2_]·2C_3_H_7_NO	*F*(000) = 1204
*M**_r_* = 1209.78	*D*_x_ = 1.529 Mg m^−^^3^
Monoclinic, *P*2_1_/*c*	Mo *K*α radiation, λ = 0.71073 Å
Hall symbol: -P 2ybc	Cell parameters from 35525 reflections
*a* = 9.2436 (5) Å	θ = 1.8–28.3°
*b* = 14.0911 (8) Å	µ = 2.09 mm^−^^1^
*c* = 20.1932 (11) Å	*T* = 298 K
β = 92.232 (1)°	Rod, red
*V* = 2628.2 (3) Å^3^	0.70 × 0.17 × 0.14 mm
*Z* = 2	

### Data collection

**Table:**

Bruker SMART APEX CCD diffractometer	6544 independent reflections
Radiation source: normal-focus sealed tube	4457 reflections with *I* > 2σ(*I*)
Graphite monochromator	*R*_int_ = 0.031
ω scans	θ_max_ = 28.3°, θ_min_ = 1.8°
Absorption correction: multi-scan (*SADABS*; Sheldrick, 1996)	*h* = −12→12
*T*_min_ = 0.323, *T*_max_ = 0.759	*k* = −18→18
35525 measured reflections	*l* = −26→26

### Refinement

**Table:**

Refinement on *F*^2^	Primary atom site location: structure-invariant direct methods
Least-squares matrix: full	Secondary atom site location: difference Fourier map
*R*[*F*^2^ > 2σ(*F*^2^)] = 0.044	Hydrogen site location: inferred from neighbouring sites
*wR*(*F*^2^) = 0.114	H-atom parameters constrained
*S* = 1.02	*w* = 1/[σ^2^(*F*_o_^2^) + (0.047*P*)^2^ + 1.9084*P*] where *P* = (*F*_o_^2^ + 2*F*_c_^2^)/3
6544 reflections	(Δ/σ)_max_ = 0.002
291 parameters	Δρ_max_ = 1.15 e Å^−^^3^
0 restraints	Δρ_min_ = −0.72 e Å^−^^3^

### Special details

**Table:**

Geometry. All e.s.d.'s (except the e.s.d. in the dihedral angle between two l.s. planes) are estimated using the full covariance matrix. The cell e.s.d.'s are taken into account individually in the estimation of e.s.d.'s in distances, angles and torsion angles; correlations between e.s.d.'s in cell parameters are only used when they are defined by crystal symmetry. An approximate (isotropic) treatment of cell e.s.d.'s is used for estimating e.s.d.'s involving l.s. planes.
Refinement. Refinement of *F*^2^ against ALL reflections. The weighted *R*-factor *wR* and goodness of fit *S* are based on *F*^2^, conventional *R*-factors *R* are based on *F*, with *F* set to zero for negative *F*^2^. The threshold expression of *F*^2^ > σ(*F*^2^) is used only for calculating *R*-factors(gt) *etc*. and is not relevant to the choice of reflections for refinement. *R*-factors based on *F*^2^ are statistically about twice as large as those based on *F*, and *R*- factors based on ALL data will be even larger.

### Fractional atomic coordinates and isotropic or equivalent isotropic displacement parameters (Å^2^)

**Table:**

	*x*	*y*	*z*	*U*_iso_*/*U*_eq_	
Cu1	0.21442 (4)	0.09166 (3)	0.42804 (2)	0.06482 (14)	
P1	0.27705 (8)	0.23843 (6)	0.40902 (4)	0.0542 (2)	
I1	0.33242 (3)	−0.04290 (2)	0.363020 (19)	0.09714 (15)	
N3	0.1824 (3)	0.0354 (2)	0.52127 (15)	0.0613 (7)	
N4	0.0105 (3)	−0.05172 (19)	0.58132 (14)	0.0566 (7)	
N5	0.5546 (5)	0.1459 (3)	0.1663 (2)	0.0951 (11)	
O1	0.7858 (7)	0.1074 (4)	0.1799 (6)	0.287 (5)	
C1	0.0525 (3)	−0.0046 (2)	0.52828 (16)	0.0516 (7)	
C2	0.1071 (4)	−0.0581 (3)	0.63210 (19)	0.0661 (9)	
H2	0.0815	−0.0900	0.6702	0.079*	
C3	0.2430 (4)	−0.0190 (3)	0.6297 (2)	0.0758 (11)	
H3	0.3091	−0.0234	0.6654	0.091*	
C4	0.2770 (4)	0.0266 (3)	0.5726 (2)	0.0756 (11)	
H4	0.3691	0.0525	0.5695	0.091*	
C5	0.4687 (3)	0.2596 (2)	0.39732 (18)	0.0581 (8)	
C6	0.5653 (4)	0.2300 (3)	0.4476 (2)	0.0754 (10)	
H6	0.5311	0.2007	0.4852	0.091*	
C7	0.7126 (4)	0.2441 (4)	0.4418 (3)	0.0928 (14)	
H7	0.7764	0.2251	0.4760	0.111*	
C8	0.7651 (4)	0.2849 (3)	0.3872 (3)	0.0953 (15)	
H8	0.8642	0.2942	0.3841	0.114*	
C9	0.6737 (5)	0.3120 (4)	0.3372 (3)	0.0958 (14)	
H9	0.7103	0.3387	0.2992	0.115*	
C10	0.5233 (4)	0.3003 (3)	0.3420 (2)	0.0790 (11)	
H10	0.4608	0.3203	0.3076	0.095*	
C11	0.1831 (3)	0.2817 (3)	0.33385 (18)	0.0634 (9)	
C12	0.1491 (4)	0.2157 (4)	0.2849 (2)	0.0829 (12)	
H12	0.1803	0.1533	0.2900	0.099*	
C13	0.0689 (6)	0.2418 (5)	0.2285 (3)	0.1131 (19)	
H13	0.0463	0.1972	0.1958	0.136*	
C14	0.0236 (6)	0.3329 (6)	0.2211 (3)	0.122 (2)	
H14	−0.0302	0.3505	0.1832	0.146*	
C15	0.0556 (6)	0.3986 (5)	0.2683 (3)	0.117 (2)	
H15	0.0232	0.4607	0.2628	0.141*	
C16	0.1362 (5)	0.3740 (4)	0.3249 (2)	0.0843 (12)	
H16	0.1589	0.4197	0.3569	0.101*	
C17	0.2317 (4)	0.3272 (3)	0.47079 (18)	0.0615 (8)	
C18	0.3051 (5)	0.4121 (3)	0.4795 (2)	0.0875 (12)	
H18	0.3856	0.4245	0.4547	0.105*	
C19	0.2605 (7)	0.4787 (4)	0.5246 (3)	0.1106 (17)	
H19	0.3113	0.5353	0.5301	0.133*	
C20	0.1431 (7)	0.4616 (4)	0.5608 (3)	0.1070 (17)	
H20	0.1131	0.5070	0.5907	0.128*	
C21	0.0690 (5)	0.3794 (5)	0.5540 (2)	0.0994 (16)	
H21	−0.0113	0.3687	0.5793	0.119*	
C22	0.1124 (4)	0.3100 (3)	0.50876 (19)	0.0756 (11)	
H22	0.0618	0.2532	0.5044	0.091*	
C23	0.6841 (10)	0.1602 (6)	0.1610 (8)	0.267 (8)	
H23	0.7095	0.2168	0.1407	0.321*	
C24	0.4410 (7)	0.2140 (5)	0.1495 (4)	0.149 (2)	
H24A	0.3958	0.2339	0.1892	0.223*	
H24B	0.3701	0.1850	0.1200	0.223*	
H24C	0.4821	0.2681	0.1283	0.223*	
C25	0.5060 (11)	0.0608 (7)	0.1946 (5)	0.216 (5)	
H25A	0.5801	0.0361	0.2244	0.324*	
H25B	0.4848	0.0154	0.1601	0.324*	
H25C	0.4202	0.0731	0.2184	0.324*	

### Atomic displacement parameters (Å^2^)

**Table:**

	*U*^11^	*U*^22^	*U*^33^	*U*^12^	*U*^13^	*U*^23^
Cu1	0.0474 (2)	0.0649 (3)	0.0820 (3)	−0.01234 (18)	0.00001 (19)	0.0069 (2)
P1	0.0421 (4)	0.0605 (5)	0.0600 (5)	−0.0076 (3)	0.0026 (3)	0.0019 (4)
I1	0.05444 (16)	0.0968 (2)	0.1387 (3)	0.00905 (13)	−0.01487 (16)	−0.03649 (19)
N3	0.0401 (13)	0.0681 (18)	0.0748 (19)	−0.0121 (12)	−0.0099 (12)	0.0072 (14)
N4	0.0402 (13)	0.0598 (16)	0.0692 (18)	−0.0035 (11)	−0.0050 (12)	0.0043 (13)
N5	0.083 (3)	0.093 (3)	0.109 (3)	−0.014 (2)	0.004 (2)	−0.006 (2)
O1	0.115 (4)	0.126 (5)	0.619 (17)	0.002 (4)	0.008 (7)	−0.049 (7)
C1	0.0363 (13)	0.0490 (17)	0.069 (2)	−0.0057 (12)	−0.0043 (13)	0.0022 (15)
C2	0.0516 (18)	0.078 (2)	0.068 (2)	−0.0042 (16)	−0.0060 (16)	0.0085 (18)
C3	0.0512 (19)	0.099 (3)	0.075 (3)	−0.0110 (19)	−0.0176 (17)	0.009 (2)
C4	0.0435 (17)	0.092 (3)	0.090 (3)	−0.0178 (17)	−0.0154 (18)	0.007 (2)
C5	0.0436 (15)	0.0559 (19)	0.075 (2)	−0.0051 (13)	0.0056 (15)	−0.0035 (16)
C6	0.0517 (19)	0.084 (3)	0.091 (3)	0.0005 (18)	0.0011 (18)	0.003 (2)
C7	0.048 (2)	0.101 (3)	0.129 (4)	0.005 (2)	−0.007 (2)	−0.002 (3)
C8	0.046 (2)	0.087 (3)	0.154 (5)	−0.005 (2)	0.016 (3)	−0.013 (3)
C9	0.068 (3)	0.099 (3)	0.123 (4)	−0.014 (2)	0.039 (3)	0.007 (3)
C10	0.059 (2)	0.092 (3)	0.087 (3)	−0.013 (2)	0.0123 (19)	0.008 (2)
C11	0.0408 (15)	0.085 (3)	0.064 (2)	−0.0089 (16)	0.0044 (14)	0.0097 (19)
C12	0.071 (2)	0.108 (3)	0.070 (3)	−0.010 (2)	−0.0067 (19)	0.000 (2)
C13	0.090 (3)	0.174 (6)	0.074 (3)	−0.019 (4)	−0.015 (3)	0.002 (4)
C14	0.078 (3)	0.199 (8)	0.087 (4)	0.006 (4)	−0.012 (3)	0.048 (5)
C15	0.097 (4)	0.145 (6)	0.110 (4)	0.031 (4)	0.016 (3)	0.054 (4)
C16	0.075 (3)	0.096 (3)	0.083 (3)	0.006 (2)	0.004 (2)	0.025 (2)
C17	0.0502 (17)	0.071 (2)	0.063 (2)	0.0037 (16)	−0.0012 (15)	0.0013 (17)
C18	0.077 (3)	0.080 (3)	0.106 (3)	−0.004 (2)	0.011 (2)	−0.019 (3)
C19	0.103 (4)	0.096 (4)	0.132 (5)	0.008 (3)	−0.003 (3)	−0.040 (3)
C20	0.102 (4)	0.115 (4)	0.104 (4)	0.032 (3)	−0.007 (3)	−0.031 (3)
C21	0.068 (3)	0.149 (5)	0.082 (3)	0.035 (3)	0.011 (2)	0.003 (3)
C22	0.0545 (19)	0.103 (3)	0.069 (2)	0.010 (2)	0.0022 (17)	−0.002 (2)
C23	0.107 (6)	0.104 (6)	0.60 (3)	−0.009 (5)	0.082 (10)	−0.038 (10)
C24	0.117 (5)	0.136 (6)	0.191 (7)	−0.015 (4)	−0.017 (5)	0.004 (5)
C25	0.198 (10)	0.212 (10)	0.236 (11)	−0.049 (8)	−0.003 (8)	0.103 (8)

### Geometric parameters (Å, º)

**Table:**

Cu1—N3	2.075 (3)	C9—H9	0.9300
Cu1—N4^i^	2.155 (3)	C10—H10	0.9300
Cu1—P1	2.1857 (10)	C11—C16	1.380 (6)
Cu1—I1	2.5731 (6)	C11—C12	1.385 (6)
P1—C5	1.821 (3)	C12—C13	1.384 (7)
P1—C11	1.824 (4)	C12—H12	0.9300
P1—C17	1.827 (4)	C13—C14	1.357 (9)
N3—C4	1.336 (5)	C13—H13	0.9300
N3—C1	1.339 (4)	C14—C15	1.354 (9)
N4—C1	1.331 (4)	C14—H14	0.9300
N4—C2	1.336 (4)	C15—C16	1.383 (7)
N4—Cu1^i^	2.155 (3)	C15—H15	0.9300
N5—C23	1.222 (8)	C16—H16	0.9300
N5—C25	1.408 (8)	C17—C18	1.383 (6)
N5—C24	1.453 (8)	C17—C22	1.388 (5)
O1—C23	1.247 (12)	C18—C19	1.381 (7)
C1—C1^i^	1.476 (6)	C18—H18	0.9300
C2—C3	1.374 (5)	C19—C20	1.354 (8)
C2—H2	0.9300	C19—H19	0.9300
C3—C4	1.367 (6)	C20—C21	1.349 (8)
C3—H3	0.9300	C20—H20	0.9300
C4—H4	0.9300	C21—C22	1.407 (7)
C5—C10	1.370 (5)	C21—H21	0.9300
C5—C6	1.390 (5)	C22—H22	0.9300
C6—C7	1.385 (5)	C23—H23	0.9300
C6—H6	0.9300	C24—H24A	0.9600
C7—C8	1.350 (7)	C24—H24B	0.9600
C7—H7	0.9300	C24—H24C	0.9600
C8—C9	1.346 (7)	C25—H25A	0.9600
C8—H8	0.9300	C25—H25B	0.9600
C9—C10	1.407 (5)	C25—H25C	0.9600
			
N3—Cu1—N4^i^	78.97 (10)	C16—C11—C12	118.5 (4)
N3—Cu1—P1	124.71 (9)	C16—C11—P1	124.2 (3)
N4^i^—Cu1—P1	119.53 (8)	C12—C11—P1	117.2 (3)
N3—Cu1—I1	105.04 (9)	C13—C12—C11	120.5 (5)
N4^i^—Cu1—I1	100.92 (8)	C13—C12—H12	119.7
P1—Cu1—I1	119.15 (3)	C11—C12—H12	119.7
C5—P1—C11	105.74 (16)	C14—C13—C12	119.7 (6)
C5—P1—C17	103.11 (16)	C14—C13—H13	120.2
C11—P1—C17	102.97 (17)	C12—C13—H13	120.2
C5—P1—Cu1	116.31 (12)	C15—C14—C13	120.8 (5)
C11—P1—Cu1	110.00 (13)	C15—C14—H14	119.6
C17—P1—Cu1	117.30 (12)	C13—C14—H14	119.6
C4—N3—C1	116.2 (3)	C14—C15—C16	120.4 (6)
C4—N3—Cu1	128.9 (2)	C14—C15—H15	119.8
C1—N3—Cu1	114.6 (2)	C16—C15—H15	119.8
C1—N4—C2	116.4 (3)	C11—C16—C15	120.1 (5)
C1—N4—Cu1^i^	111.74 (19)	C11—C16—H16	119.9
C2—N4—Cu1^i^	131.5 (2)	C15—C16—H16	119.9
C23—N5—C25	120.3 (8)	C18—C17—C22	118.5 (4)
C23—N5—C24	124.8 (7)	C18—C17—P1	123.7 (3)
C25—N5—C24	114.7 (6)	C22—C17—P1	117.7 (3)
N4—C1—N3	125.9 (3)	C19—C18—C17	120.9 (5)
N4—C1—C1^i^	117.6 (3)	C19—C18—H18	119.5
N3—C1—C1^i^	116.5 (4)	C17—C18—H18	119.5
N4—C2—C3	122.0 (4)	C20—C19—C18	120.0 (5)
N4—C2—H2	119.0	C20—C19—H19	120.0
C3—C2—H2	119.0	C18—C19—H19	120.0
C4—C3—C2	117.3 (3)	C21—C20—C19	120.8 (5)
C4—C3—H3	121.3	C21—C20—H20	119.6
C2—C3—H3	121.3	C19—C20—H20	119.6
N3—C4—C3	122.2 (3)	C20—C21—C22	120.4 (5)
N3—C4—H4	118.9	C20—C21—H21	119.8
C3—C4—H4	118.9	C22—C21—H21	119.8
C10—C5—C6	118.4 (3)	C17—C22—C21	119.3 (5)
C10—C5—P1	124.4 (3)	C17—C22—H22	120.4
C6—C5—P1	117.2 (3)	C21—C22—H22	120.4
C7—C6—C5	120.0 (4)	N5—C23—O1	127.1 (10)
C7—C6—H6	120.0	N5—C23—H23	116.4
C5—C6—H6	120.0	O1—C23—H23	116.4
C8—C7—C6	121.1 (4)	N5—C24—H24A	109.5
C8—C7—H7	119.5	N5—C24—H24B	109.5
C6—C7—H7	119.5	H24A—C24—H24B	109.5
C9—C8—C7	119.9 (4)	N5—C24—H24C	109.5
C9—C8—H8	120.1	H24A—C24—H24C	109.5
C7—C8—H8	120.1	H24B—C24—H24C	109.5
C8—C9—C10	120.5 (4)	N5—C25—H25A	109.5
C8—C9—H9	119.7	N5—C25—H25B	109.5
C10—C9—H9	119.7	H25A—C25—H25B	109.5
C5—C10—C9	120.1 (4)	N5—C25—H25C	109.5
C5—C10—H10	120.0	H25A—C25—H25C	109.5
C9—C10—H10	120.0	H25B—C25—H25C	109.5

Symmetry code: (i) −*x*, −*y*, −*z*+1.

### Hydrogen-bond geometry (Å, º)

**Table:**

*D*—H···*A*	*D*—H	H···*A*	*D*···*A*	*D*—H···*A*
C4—H4···I1^ii^	0.93	3.03	3.796 (4)	140

Symmetry code: (ii) −*x*+1, −*y*, −*z*+1.
